# Teaching Methodologies and Educational Outcomes of Point‐of‐Care Ultrasound in Undergraduate Medical Education: A Scoping Review

**DOI:** 10.1111/tct.70485

**Published:** 2026-07-29

**Authors:** Isabel Dutra da Cruz, Marlon Natan Baracho de Oliveira, Alessandra Mazzo, Raphael Raniere de Oliveira Costa

**Affiliations:** ^1^ Escola Multicampi de Ciências Médicas Universidade Federal do Rio Grande do Norte (UFRN) Caicó RN Brazil; ^2^ Faculdade de Medicina de Bauru Universidade de São Paulo (USP) Bauru SP Brazil

**Keywords:** educational technology, medical education, medical students, teaching, ultrasound

## Abstract

**Objective:**

This study aimed to identify the teaching methodologies used in point‐of‐care ultrasound (POCUS) education for undergraduate medical students and to synthesise the educational outcomes associated with these approaches.

**Methods:**

A scoping review followed Joanna Briggs Institute methodology and Preferred Reporting Items for Systematic Reviews and Meta‐Analyses extension for Scoping Reviews (PRISMA‐ScR) guidelines. The research question was developed using the Population–Concept–Context framework. Systematic searches were performed in PubMed/MEDLINE, Web of Science, Scopus, Embase, CINAHL and ERIC. The review protocol was prospectively registered in the Open Science Framework.

**Results:**

Fifty‐nine studies were included. Teaching methodologies comprised supervised practice with real patients, simulation using high‐fidelity and low‐cost models, didactic lectures, active learning strategies (including flipped classroom and peer‐assisted learning), short and longitudinal courses and educational technologies such as e‐learning platforms, portable ultrasound devices and virtual reality. Assessment strategies included objective structured clinical examinations, structured questionnaires and theoretical and practical tests. The most frequently reported educational outcomes were improved diagnostic accuracy and clinical performance, technology‐supported theoretical learning, increased confidence and practical autonomy and the educational benefits of integrated active learning approaches. Educational interventions combining supervised practice, simulation and active learning were consistently associated with more favourable educational outcomes than isolated teaching strategies.

**Conclusions:**

The structured and longitudinal integration of POCUS into undergraduate medical curricula appears to be a promising strategy for strengthening clinical reasoning, diagnostic accuracy and bedside decision‐making. These findings support implementing competency‐based curricula that integrate supervised practice, simulation and active learning methodologies, while highlighting the importance of longitudinal curricular integration for sustainable competency development.

## 1 Introduction

Point‐of‐care ultrasound (POCUS), or bedside ultrasonography, has been established as a rapid, safe and noninvasive diagnostic tool, widely used in contemporary clinical practice. Broadly, it is defined as the acquisition, interpretation and immediate clinical integration of ultrasonographic images performed by the attending physician in the context of direct patient care [1]. More than the place where it is performed or the type of equipment used, POCUS is characterised by its immediate clinical application, supporting diagnostic and therapeutic decision‐making in real time.

In clinical practice, its use has expanded across different settings, especially in urgent care, emergency, and intensive care, in which rapid decision‐making is essential. The use of POCUS enables dynamic assessment of organs such as the lungs, heart and abdominal cavity, reducing the need for more time‐consuming or invasive complementary examinations. In addition, its application in image‐guided procedures, such as vascular access, contributes to greater safety and a reduction in complications, reinforcing its clinical relevance and impact on patient outcomes [2].

Given this growing incorporation into medical practice, the adequate training of future professionals becomes a central element to ensure the safe, effective and evidence‐based use of this technology. The inclusion of POCUS in undergraduate and medical residency curricula, with emphasis on the physical principles of ultrasound, image acquisition and interpretation and its integration into clinical reasoning, has been pointed out as indispensable for the development of contemporary competencies in medicine [3].

However, the implementation of POCUS teaching still faces important challenges. Among the main obstacles are the lack of curricular standardisation, the shortage of qualified faculty and the heterogeneity of the pedagogical strategies used among institutions. Such gaps may compromise the acquisition of essential competencies, generate insecurity in image acquisition and interpretation and consequently, impact diagnostic accuracy and clinical decision‐making [4].

In this context, different pedagogical methodologies have been employed for POCUS teaching, including lectures, digital resources, active methodologies, clinical simulation and supervised bedside practice. Structured strategies, such as specific frameworks for ultrasonographic assessment and progressive models based on skill levels, have demonstrated potential to organise the teaching‐learning process and favour the safe development of clinical competencies. Additionally, immediate image review and structured feedback have been identified as fundamental elements for the consolidation of learning [5].

Recent studies have demonstrated that even short‐duration educational interventions, when carefully planned, can promote significant improvements in students' cognitive and technical performance, particularly when combining theoretical content with supervised hands‐on training [6]. However, important disparities persist in the way these strategies are implemented, both among institutions and across different stages of medical training.

Despite the growing international interest in the incorporation of POCUS into medical education, the description of the pedagogical methodologies used and their educational outcomes remains heterogeneous. Although studies are addressing specific curricular experiences, it is still not clearly synthesised which teaching strategies have been most frequently used and which outcomes have been observed in the training of undergraduate medical students.

To our knowledge, no previous scoping review has comprehensively synthesised both teaching methodologies and educational outcomes in undergraduate POCUS education. In view of this scenario, this study aims to identify the methodologies used in POCUS teaching and their outcomes for undergraduate medical students.

## 2 Methods

This is a scoping review conducted in accordance with the methodology proposed by the Joanna Briggs Institute (JBI) for evidence synthesis and reported following the Preferred Reporting Items for Systematic Reviews and Meta‐Analyses extension for Scoping Reviews (PRISMA‐ScR) [7, 8].

The review was developed according to the stages recommended by the JBI: definition of the objective and research question; establishment of eligibility criteria; planning of the search strategy, selection, data extraction and data synthesis; systematic search in the selected databases; screening and selection of studies; data extraction; synthesis and analysis of the evidence; and structured presentation of the results [8].

For the formulation of the guiding research question, the Population, Concept, and Context (PCC) strategy was used, as recommended by the JBI. The following elements were defined: P (Population)—undergraduate medical students, C (Concept)—teaching methodologies for POCUS and C (Context)—the context of undergraduate medical education. Based on this framework, the following research question was developed: What POCUS teaching methodologies are used in undergraduate medical education, and what are their main educational outcomes?

The search strategy was developed with the support of a specialist in the field and subsequently registered on the Open Science Framework (OSF) platform under DOI: 10.17605/OSF.IO/WCB5P.

The systematic search was carried out on 15 May 2025, in the following databases: PubMed/MEDLINE, Web of Science, Scopus, Embase, CINAHL and ERIC. To construct the search strategy, descriptors from the DeCS and MeSH systems were used, in addition to free keywords, synonyms and terms related to the components of the PCC strategy. The descriptors were combined using the Boolean operators AND, OR, with specific adaptations for each database. The complete search strategy was made available in Appendix S1.

Studies published in scientific journals, with no language or publication period restrictions, addressing POCUS teaching methodologies and their educational outcomes among undergraduate medical students, were included. Book chapters, theses, dissertations, conference abstracts, editorials and studies that did not answer the research question were excluded.

**FIGURE 1:**
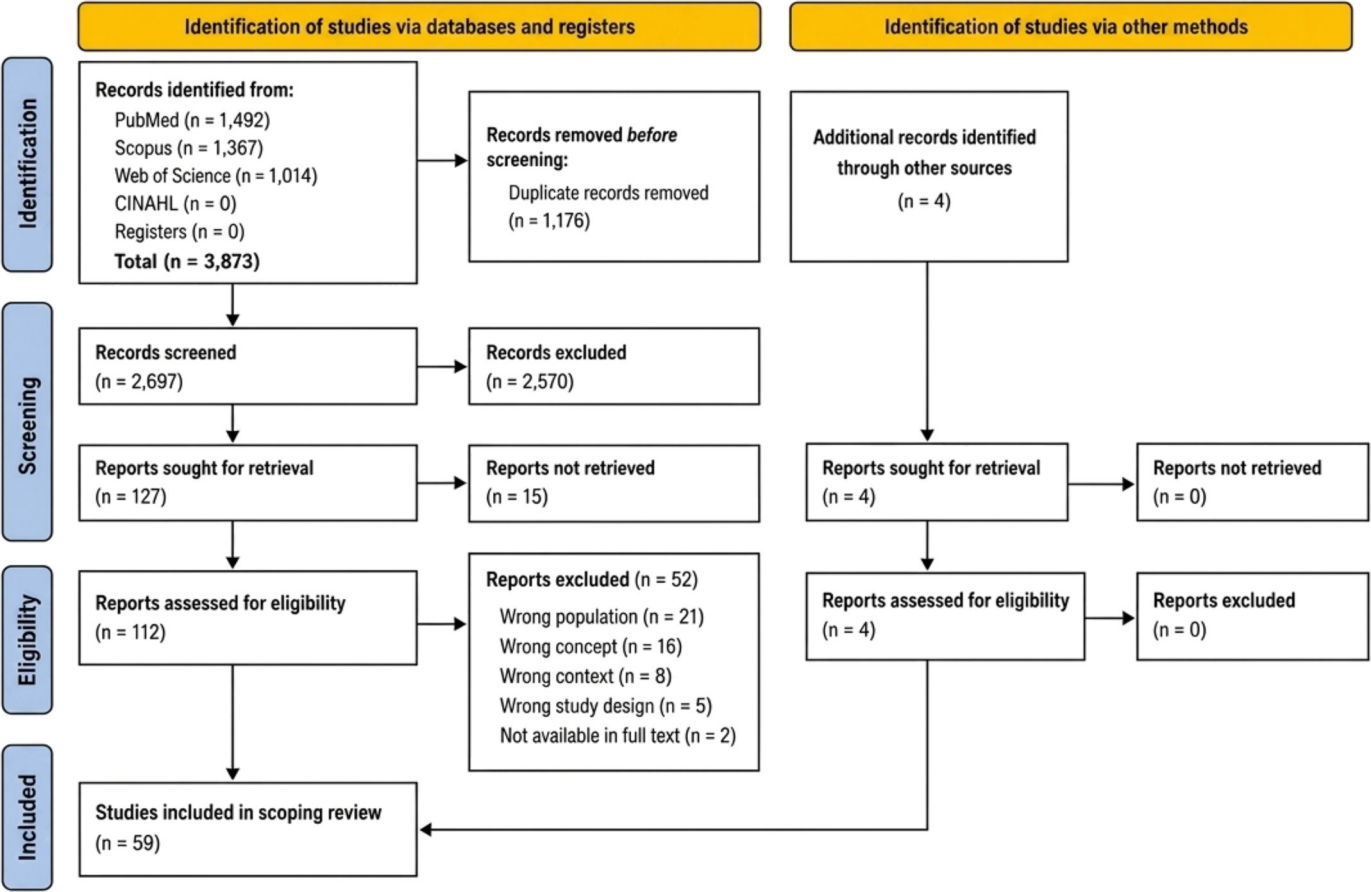
PRISMA 2020 flow diagram of the study selection process.

Initially, 220 records were identified through database searching. After removing seven duplicate records, 214 records underwent title and abstract screening. Of these, 87 reports were assessed for eligibility through full‐text review. Following the identification and removal of one duplicate study during the revision process, 59 unique studies were included in the final scoping review. The study selection process is presented in the PRISMA‐ScR flow diagram (Figure 1).

All screening and selection stages were performed by two independent reviewers, in accordance with JBI methodological recommendations, with disagreements resolved by consensus.

For data extraction, a standardised instrument was used, including the following variables: author, year of publication, country, study population, methodological design, teaching methodology employed, assessment instruments and main educational outcomes.

Subsequently, the data were subjected to descriptive thematic synthesis, allowing the findings to be grouped into categories related to pedagogical methodologies and observed educational outcomes, with subsequent presentation in tables and figures. This approach enabled the mapping of the available evidence, the identification of methodological trends, and the analysis of the main outcomes in medical education [9].

Data extraction was independently performed by two reviewers using a standardised data extraction form developed for this review. The extraction instrument was piloted on a sample of included studies to ensure consistency and completeness before full data extraction. Any discrepancies were resolved through discussion until consensus was reached.

To facilitate interpretation of the evidence, an interpretative matrix relating teaching methodologies to educational outcomes was developed. Ratings of *High*, *Moderate* and *Low* were assigned through qualitative thematic synthesis based on the consistency and recurrence of evidence across the included studies. A *High* rating indicated that a methodology was consistently associated with a given outcome across multiple studies; *Moderate* indicated recurring but less consistent evidence, and *Low* indicated limited or infrequently reported evidence. Two reviewers independently proposed the classifications, which were subsequently refined through discussion until consensus was achieved. These ratings represent qualitative summaries of the mapped evidence and should not be interpreted as comparative measures of educational effectiveness.

As this study was based on secondary data in the public domain, submission to the Research Ethics Committee was not required.

## 3 Results

**FIGURE 2:**
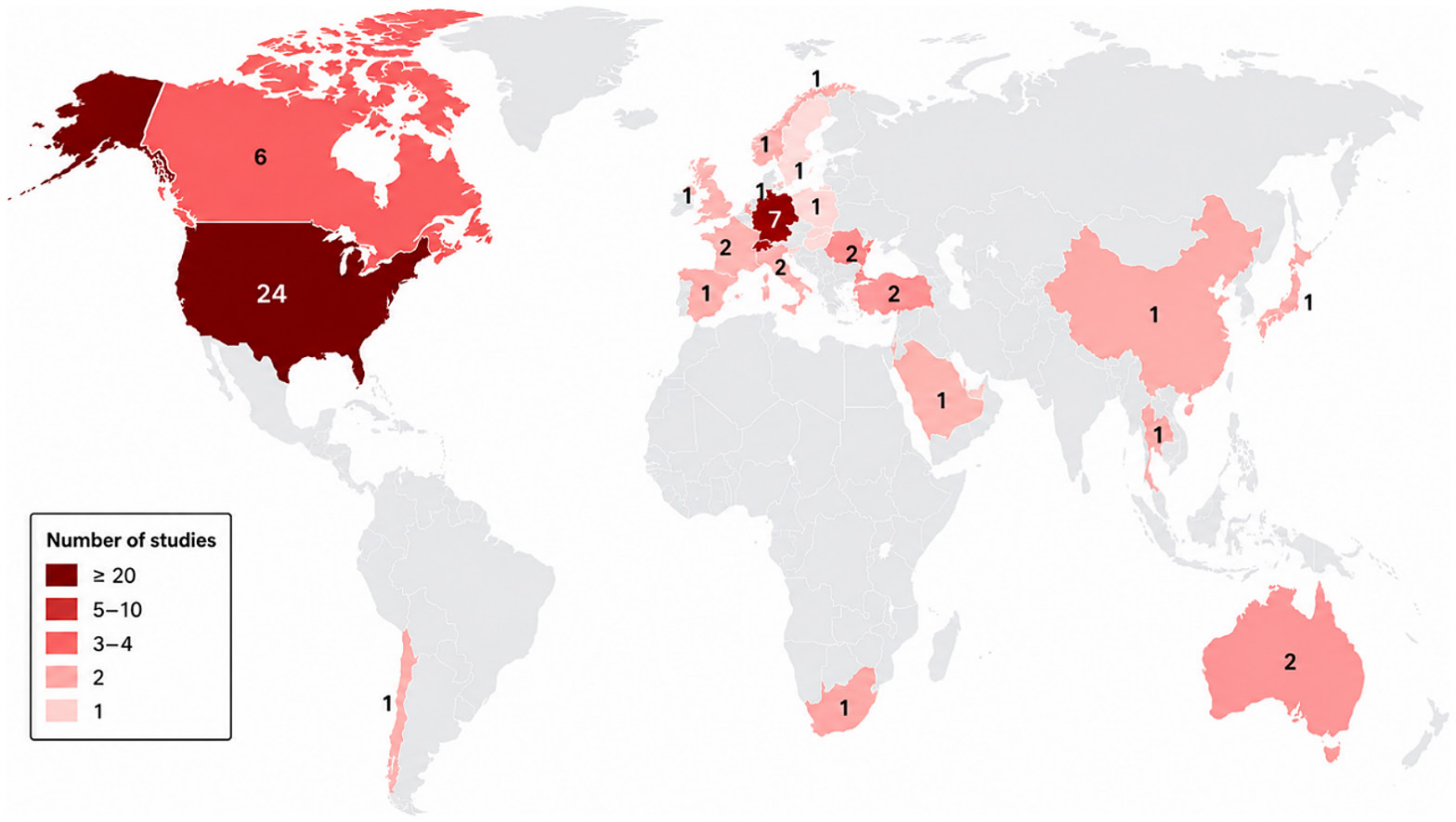
Geographic distribution of included studies (*n* = 59). *Note:* Numbers indicate the number of studies included in each country. Colour intensity represents the frequency of studies. The United States (*n* = 24), Germany (*n* = 7) and Canada (*n* = 6) accounted for the largest proportions of included studies. The total number of studies included was 59.

Among the 59 studies included in this scoping review, 25 (42.4%) were published within the last 5 years, demonstrating the growing interest in POCUS education in undergraduate medical training. Most studies were conducted in North America (*n* = 30; 50.8%), followed by Europe, Asia, South America and Oceania, reflecting the global expansion of POCUS curricula. Figure 2 presents the geographical distribution of the included studies.

World map showing the countries represented in the studies was included in this scoping review. Numbers indicate the frequency of studies conducted in each country, and darker shades represent a higher number of publications. The United States (*n* = 24), Germany (*n* = 7) and Canada (*n* = 6) accounted for the largest proportion of studies (Figure 2).

Review articles (narrative reviews, systematic reviews, and scoping reviews) were included to map how POCUS teaching methodologies and educational outcomes have been synthesised in the literature. Data were extracted only from the included review articles themselves. Primary studies cited within these reviews were not re‐extracted or counted separately in the frequency analyses presented in Tables 1 and 2, thereby avoiding double‐counting of evidence.

**TABLE 1:** Teaching methodologies identified in undergraduate POCUS education (*n* = 59).

Teaching methodology category	Teaching methodology	Frequency (*n*)
Didactic teaching	Didactic lectures	10
Supervised practical training	Real patient practice	12
	High‐fidelity simulation	6
	Low‐cost handmade models	6
	**Subtotal**	**24**
Assessment strategies	Objective structured clinical examination (OSCE)	8
	Structured questionnaires	8
	Theoretical and practical tests (pre/post)	8
	**Subtotal**	**24**
Active learning methodologies	Flipped classroom	7
	Peer‐assisted learning	4
	Gamification	4
	**Subtotal**	**15**
Courses and curriculum models	Short courses (1 day to 4 weeks)	9
	Longitudinal curricula	5
	**Subtotal**	**14**
Educational technologies	E‐learning platforms	6
	Portable ultrasound devices	5
	Virtual reality	3
	**Subtotal**	**14**

*Note:* Frequencies represent the number of times each teaching methodology was reported across the included studies. Because individual studies frequently incorporated multiple educational approaches, frequencies are not mutually exclusive and should not be interpreted as the number of unique studies.

**TABLE 2:** Educational outcomes reported in undergraduate POCUS education (*n* = 59).

Educational outcome	Summary of evidence	Studies (*n*)
Improved diagnostic accuracy and clinical performance	Improvements in image acquisition, interpretation, procedural performance and diagnostic accuracy following practical POCUS training.	10
Technology‐supported theoretical learning	E‐learning, podcasts, videos and digital resources enhanced theoretical learning and content retention.	10
Increased confidence and practical autonomy	Practical training increased students' confidence, self‐efficacy and readiness to perform POCUS examinations.	9
Benefits of active and combined educational methodologies	Flipped classroom, peer‐assisted learning and integrated curricula produced superior educational outcomes when combined with hands‐on practice.	9
Student engagement and satisfaction	High levels of satisfaction, motivation and engagement with POCUS learning activities.	8
Feasibility and sustainability	Educational models were considered feasible across diverse curricular contexts.	7
Knowledge retention	Longitudinal curricula and repeated practice improved retention of knowledge and skills.	6
Implementation barriers	Faculty availability, curricular integration, infrastructure and time constraints limited implementation.	6
Importance of feedback and supervised practice	Continuous supervision and structured feedback enhanced competency acquisition.	5
Long‐term transfer of learning	Evidence supporting long‐term transfer to clinical practice remained limited.	4

*Note:* Frequency indicates the number of studies included reporting each educational outcome. Individual studies frequently contributed to more than one outcome category; therefore, frequencies are not mutually exclusive.

All included studies were conducted within the field of medical education. The study populations primarily consisted of undergraduate medical students from different stages of training, ranging from preclinical to internship years. A small number of studies included mixed populations comprising residents, faculty members, radiologists or clinical educators involved in POCUS training and curriculum development.

Regarding methodological design, substantial heterogeneity was observed. The included evidence comprised cross‐sectional studies, prospective observational studies, quasi‐experimental studies, randomised controlled trials, qualitative studies, narrative reviews, systematic reviews and scoping reviews. Quasi‐experimental and observational quantitative studies predominated, reflecting the emphasis on evaluating educational interventions and learner outcomes. Detailed characteristics of the included studies are presented in the Table S1.

### 3.1 Teaching Methodologies Identified

The analysis of the evidence allowed the identification of a wide diversity of pedagogical strategies employed in POCUS teaching. The analysis of the evidence identified a wide range of pedagogical strategies used in undergraduate POCUS education. These strategies were grouped inductively into six categories: didactic lectures, supervised practical training, assessment strategies, active learning methodologies, courses and curriculum models and educational technologies (Table 2).

Among the identified methodologies, supervised practice (*n* = 24) stood out as the most frequent strategy, with a predominance of the use of real patients (*n* = 12), followed by high‐fidelity simulators (*n* = 6) and low‐cost handmade models (*n* = 6). This predominance suggests a strong emphasis on experiential learning and the progressive development of psychomotor skills in POCUS education (Table 1).

Assessment strategies also showed high frequency (*n* = 24), especially through OSCEs (*n* = 8), structured questionnaires (*n* = 8) and theoretical/practical pre‐ and post‐intervention tests (*n* = 8), indicating a consistent concern with the objective measurement of clinical competencies.

Active methodologies were widely represented (*n* = 15), with emphasis on the flipped classroom (*n* = 7), followed by peer‐assisted learning (PAL) and gamification. An important presence of educational technologies was also observed (*n* = 14), including portable devices, digital platforms and virtual reality resources, evidencing the increasing incorporation of digital tools into medical education.

Overall, the studies revealed a tendency toward the adoption of hybrid pedagogical models, combining theory, supervised practice, technological resources and student‐centred methodologies.

### 3.2 Main Educational Outcomes

The main educational outcomes were grouped into 10 thematic categories encompassing clinical performance, student confidence, skill retention, implementation challenges and the feasibility of educational models (Table 2).

The most frequently reported educational outcomes were improved diagnostic accuracy and clinical performance (*n* = 10), effectiveness of technology‐supported theoretical teaching (*n* = 10), increased confidence and practical autonomy (*n* = 9) and positive outcomes associated with active and combined educational approaches (*n* = 9). Collectively, these findings suggest that educational strategies integrating supervised practice, simulation, active learning methodologies and technology‐enhanced instruction were more frequently associated with positive educational outcomes than isolated approaches.

The findings consistently indicated positive effects on learners' confidence, practical autonomy, diagnostic accuracy and clinical performance. Educational approaches combining supervised practice, simulation and authentic clinical exposure were most frequently associated with improvements in technical competence and clinical reasoning. In contrast, evidence regarding long‐term skill retention was less frequently reported and was primarily observed in studies evaluating longitudinal curricula, highlighting the potential importance of sustained educational exposure for competency maintenance.

Technological tools, such as e‐learning platforms, narrated videos, podcasts and flipped classroom approaches, proved effective in supporting theoretical knowledge acquisition. However, several studies highlighted limitations in the transfer of theoretical knowledge to practical scanning skills when these strategies were not accompanied by structured supervision and hands‐on training.

Another recurrent finding was the high level of student acceptance and engagement associated with innovative educational approaches, particularly gamification, PAL, workshops and hybrid curricula. Nevertheless, important implementation challenges remained, including infrastructure constraints, limited faculty availability, lack of standardisation and difficulties in achieving longitudinal curricular integration of POCUS within undergraduate medical education.

Overall, the findings suggest that integrated pedagogical models based on supervised practice, simulation, active learning and technology‐enhanced instruction may provide broader educational benefits than isolated approaches, particularly in the development of applied clinical competencies.

The classifications presented in Table 3 represent an interpretative synthesis of the educational outcomes reported across the included studies and should not be interpreted as comparative measures of effectiveness.

**TABLE 3:** Relationship between teaching methodologies and educational outcomes in undergraduate POCUS education.

Teaching methodology	Knowledge acquisition	Practical skills	Confidence/self‐efficacy	Diagnostic performance	Learner satisfaction	Skill retention
Didactic lectures	High	Low	Low	Low	Moderate	Low
Real patient practice	Moderate	High	High	High	Moderate	Moderate
High‐fidelity simulation	Moderate	High	Moderate	Moderate	Moderate	Moderate
Low‐cost simulation models	Moderate	High	Moderate	Moderate	Moderate	Low
Flipped classroom	High	Moderate	Moderate	Low	High	Moderate
Peer‐assisted learning	Moderate	Moderate	Moderate	Low	High	Moderate
Gamification	Moderate	Moderate	Moderate	Low	High	Low
E‐learning	High	Low	Low	Low	Moderate	Moderate
Portable ultrasound devices	Moderate	Moderate	Moderate	Low	Moderate	Moderate
Virtual reality	Moderate	Moderate	Moderate	Low	Moderate	Low
Short courses	Moderate	Moderate	Moderate	Low	Moderate	Low
Longitudinal curricula	High	High	High	Moderate	Moderate	High

*Note:* Ratings represent an interpretative synthesis of the evidence reported across the included studies. Classifications were assigned through qualitative thematic synthesis performed independently by the reviewers and finalised by consensus. High indicates consistent evidence reported across multiple studies, moderate indicates recurring but less consistent evidence and low indicates limited or infrequently reported evidence. These ratings should be interpreted as qualitative summaries rather than comparative measures of effectiveness.

Across the methodologies analysed, hybrid approaches demonstrated the broadest range of positive educational outcomes, being consistently associated with gains in knowledge acquisition, practical skills, confidence, diagnostic accuracy and student satisfaction. Experiential strategies, including real patient practice, simulation and longitudinal curricula, were more strongly associated with the development of technical competence, clinical reasoning and learner autonomy. Conversely, technology‐based approaches such as e‐learning and flipped classroom models were predominantly linked to theoretical knowledge acquisition. However, their impact on practical skills appeared more limited when implemented without supervised clinical practice. These findings reinforce the value of multimodal and longitudinal educational models for competency development in undergraduate POCUS education.

## 4 Discussion

This scoping review aimed to identify and synthesise the teaching methodologies used in POCUS education for undergraduate medical students and to map the educational outcomes associated with these approaches. Overall, the findings indicate that diverse pedagogical strategies—including interactive lectures, supervised practice, active learning methodologies, educational technologies and longitudinal curricula—are consistently associated with improvements in clinical reasoning, diagnostic accuracy, decision‐making, learner confidence and engagement. These findings are consistent with previous reviews demonstrating the educational value of POCUS in undergraduate medical education, while providing an updated synthesis specifically focused on the relationship between teaching methodologies and their educational outcomes [10, 11, 12, 13].

*Overall, the findings indicate that diverse pedagogical strategies—including interactive lectures, supervised practice, active learning methodologies, educational technologies and longitudinal curricula—are consistently associated with improvements in clinical reasoning, diagnostic accuracy, decision‐making, learner confidence, and engagement*.

The temporal distribution of the included studies illustrates the maturation of undergraduate POCUS education over the past two decades. Earlier publications primarily focused on demonstrating the feasibility of curricular integration, whereas studies published during the last 5 years increasingly examined educational effectiveness, competency development, learner engagement and longitudinal curriculum design. This evolution mirrors the trajectory described by Birrane et al. [11], who characterised the initial expansion of undergraduate ultrasound curricula as largely centred on implementation and feasibility. More recent reviews have similarly highlighted the transition toward competency‐based and longitudinal educational models [12, 13]. Our findings extend this evidence by demonstrating that contemporary research increasingly evaluates not only curriculum implementation but also clinically meaningful educational outcomes, including diagnostic reasoning, clinical decision‐making and knowledge retention.

The predominance of studies conducted in North America reflects the earlier adoption and consolidation of POCUS within undergraduate curricula, likely influenced by international consensus statements and specialty‐specific recommendations supporting ultrasound competency in medical education [14]. Nevertheless, increasing contributions from Europe, Asia, South America and Oceania indicate that undergraduate POCUS education is becoming progressively more international, although important geographical disparities in curricular implementation and educational resources remain.

The analysed studies demonstrated that POCUS teaching is implemented across different phases of undergraduate medical education, with greater concentration during the first 4 years of training. This finding reinforces the concept of POCUS as a transversal clinical competency that allows students to develop image‐based reasoning and anatomical‐functional integration from the early stages of medical education. Successful curricular models, such as those implemented at Harvard Medical School, demonstrate that longitudinal integration, progressive increases in complexity and interdisciplinary collaboration may facilitate sustainable implementation throughout undergraduate training [10]. Similar recommendations have been consistently reported in previous reviews, which advocate for longitudinal curricula rather than isolated workshops or elective experiences [11, 12, 13].

Among the identified methodologies, supervised hands‐on practice emerged as the most frequently employed educational strategy, particularly through the use of real patients, high‐fidelity simulators and low‐cost handmade models. This predominance highlights the central role of experiential learning in POCUS education, as competency development requires the integration of psychomotor skills, real‐time image interpretation and clinical reasoning. The combination of simulation with supervised clinical exposure enables deliberate practice in safe learning environments while progressively increasing clinical complexity [15, 16, 17]. These findings corroborate those reported by Birrane et al. [11], who identified practical training as the cornerstone of undergraduate ultrasound education. However, the present review demonstrates a broader incorporation of simulation‐based approaches, low‐cost educational resources and authentic clinical experiences, reflecting increasing pedagogical sophistication and greater concern with scalability across diverse educational contexts.

Assessment strategies also represented a prominent component of undergraduate POCUS curricula. Objective structured clinical examinations (OSCEs), theoretical‐practical examinations, structured checklists and retention assessments were frequently employed, often accompanied by immediate feedback and formative assessment. These approaches support the consolidation of technical performance while fostering confidence and clinical reasoning. Nevertheless, these findings should be interpreted alongside those reported by DeBiasio et al. [10], whose scoping review specifically examined assessment methods in undergraduate POCUS education. Those authors concluded that assessment remains predominantly focused on technical performance and image acquisition, with limited evaluation of higher‐order competencies corresponding to the upper levels of Miller's pyramid. Although technical assessments continued to predominate among the studies included in the present review, we identified an increasing incorporation of formative feedback, longitudinal follow‐up and structured competency assessments, suggesting a gradual shift toward more comprehensive competency‐based evaluation.

Active learning methodologies—including flipped classroom, PAL, and gamification—were consistently associated with improved learner engagement, autonomy, and knowledge retention. Flipped classroom approaches appeared particularly effective by reserving face‐to‐face time for supervised scanning and clinical application while delivering conceptual content through virtual learning environments [18]. PAL likewise expanded practical training opportunities while reducing dependence on expert instructors. Although Birrane et al. [11] previously identified peer teaching and educational adjuncts as promising strategies, our review demonstrates a considerably broader adoption of contemporary active learning methodologies, reflecting ongoing educational innovation within undergraduate medical education.

Educational technologies likewise emerged as central components of contemporary POCUS education. Portable ultrasound devices, e‐learning platforms, mobile applications, artificial intelligence‐assisted resources and immersive technologies such as virtual reality were increasingly incorporated into undergraduate curricula. These technologies appear to enhance flexibility, facilitate repeated practice, expand access to educational opportunities and provide immediate learner feedback [19]. McCormick et al. [12] similarly highlighted the educational value of portable ultrasound devices in undergraduate education. Our findings extend these observations by demonstrating the growing diversification of digital educational resources, reflecting the broader digital transformation currently occurring in health professions education.

Despite these positive developments, important challenges remain for the widespread implementation of undergraduate POCUS curricula. Previous reviews consistently identify barriers including limited faculty expertise, equipment costs, curriculum time constraints and logistical constraints associated with practical teaching [12, 13]. Similar challenges were reflected across the studies included in the present review. Although resource limitations were not directly investigated by the studies included, educational inequities remain an important contextual consideration, particularly in low‐ and middle‐income countries. In Brazil, for example, unequal distribution of ultrasound equipment and trained faculty between regions and institutions may further limit students' opportunities for practical training. This observation should be interpreted as contextual information rather than as a finding generated by the present review, but it reinforces the importance of adaptable curricular models capable of accommodating different institutional realities.

Collectively, these findings reinforce the need for competency‐based longitudinal curricula integrating theoretical instruction, simulation, supervised clinical practice, educational technologies and formative assessment. Such curricular structures may facilitate not only the acquisition of technical skills but also their retention and progressive integration into clinical reasoning. While previous reviews have emphasised the feasibility of curricular implementation [11], national experiences of curriculum integration [12], assessment methods [10] or broad recommendations for curriculum development [13], the present scoping review contributes a complementary perspective by explicitly mapping teaching methodologies alongside their associated educational outcomes. This synthesis offers educators a broader understanding of how different instructional approaches contribute to competency development and may inform curriculum planning across diverse educational settings.

*Collectively, these findings reinforce the need for competency‐based longitudinal curricula integrating theoretical instruction, simulation, supervised clinical practice, educational technologies and formative assessment*.

### 4.1 Practice Implications

From a practical perspective, undergraduate POCUS curricula should prioritise longitudinal integration across multiple years of medical training rather than isolated educational interventions. Practical sessions should combine supervised hands‐on scanning with simulation and structured formative feedback to promote progressive competency development. Active learning strategies, including flipped classroom and PAL, may maximise learner engagement while optimizing faculty resources. For institutions with limited infrastructure, portable ultrasound devices, low‐cost simulation models, blended learning and peer‐assisted teaching represent feasible alternatives that may substantially reduce logistical barriers without compromising educational quality. These approaches may facilitate broader implementation across institutions with different economic, technological and organisational contexts.

The findings should be interpreted considering several limitations. The methodological heterogeneity of the included studies—including differences in curricular structures, educational interventions, assessment methods and reported outcomes—limited direct comparisons across studies. Most investigations evaluated short‐term educational outcomes, whereas evidence regarding long‐term competency retention, transfer of learning to clinical practice and patient‐related outcomes remains limited. Furthermore, the predominance of studies conducted in North America may limit the generalizability of findings to educational systems with different curricular structures and resource availability. Finally, as expected in a scoping review, no formal appraisal of methodological quality or risk of bias was performed, limiting conclusions regarding the comparative effectiveness of individual educational interventions.

Future research should prioritise multicentre longitudinal studies capable of evaluating competency retention, transfer of learning into clinical practice, patient‐related outcomes and implementation strategies across diverse educational contexts. Greater standardisation of curricular competencies and assessment frameworks may also facilitate comparisons between institutions and contribute to the development of internationally applicable recommendations for undergraduate POCUS education.

## 5 Conclusion

This scoping review synthesised the teaching methodologies used in POCUS education for undergraduate medical students and the educational outcomes associated with these approaches.

The findings suggest that educational strategies integrating supervised practice, simulation, active learning methodologies and educational technologies are more frequently associated with positive outcomes, particularly improvements in diagnostic accuracy, clinical performance, learner confidence and engagement. Competency‐based and longitudinal curricular models appear promising for supporting the integration of POCUS into undergraduate medical education.

*Competency‐based and longitudinal curricular models appear promising for supporting the integration of POCUS into undergraduate medical education*.

The findings suggest that educational strategies integrating supervised practice, simulation, active learning methodologies and educational technologies are more frequently associated with positive outcomes, particularly improvements in diagnostic accuracy, clinical performance, learner confidence and engagement.

Despite these positive findings, important challenges remain related to curricular standardisation, infrastructure, faculty development, and programme sustainability. Additionally, evidence regarding long‐term skill retention remains limited. Future studies should prioritise longitudinal evaluations examining competency retention and the transfer of learning to clinical practice.

## Author Contributions

**Isabel Dutra da Cruz:** conceptualization, investigation, writing – original draft, writing – review and editing, visualization, validation, methodology, formal analysis, data curation. **Marlon Natan Baracho de Oliveira:** writing – original draft, writing – review and editing, validation, visualization, data curation. **Alessandra Mazzo:** writing – original draft, writing – review and editing, visualization, validation. **Raphael Raniere de Oliveira Costa:** conceptualization, investigation, writing – original draft, supervision, data curation, formal analysis, project administration, writing – review and editing, visualization, validation, methodology.

## Funding

This study was financed in part by the Coordenação de Aperfeiçoamento de Pessoal de Nível Superior—Brasil (CAPES)—Finance Code 001.

## Disclosure

The views expressed in this manuscript are those of the authors and do not necessarily represent the official position of the affiliated institution.

## Conflicts of Interest

The authors declare no conflicts of interest.

## Supporting information


**Appendix S1:** Supporting information.

**Table S1:** Characteristics of included studies on point‐of‐care ultrasound (POCUS) education in undergraduate medical education (*n* = 59).

## Data Availability

The data that support the findings of this study are available from the corresponding author upon reasonable request.
