# Supplementary material for: Teaching Methodologies and Educational Outcomes of Point‐of‐Care Ultrasound in Undergraduate Medical Education: A Scoping Review
Source: Clin Teach. 2026 Jul 29;23(5):e70485. doi: 10.1111/tct.70485 (PMC13420744; doi:10.1111/tct.70485)
Supplement: Supplementary file 1 — Appendix S1: Supporting information. [file TCT-23-e70485-s001.docx]

# Supplementary Appendix S1

**Complete Electronic Search Strategies**

The electronic search strategies were developed according to the Population–Concept–Context (PCC) framework recommended by the Joanna Briggs Institute. Searches were conducted on May 15, 2025, and were adapted to the controlled vocabulary, indexing terms, and search syntax of each database.

## PubMed/MEDLINE

(point of care systems OR point of care AND systems OR point of care OR point AND care
OR diagnostic imaging OR diagnostic AND imaging
OR ultrasound OR ultrasonography OR ultrasonics OR ultrasounds)

AND

(Academic Training OR Activities, Training OR Activity, Training OR Educational Technic
OR Educational Technics OR Educational Technique OR Educational Techniques
OR Method, Teaching OR Methods, Teaching OR Pedagogies OR Pedagogy
OR Teaching Method OR Teaching Methods OR Teaching-Learning Process
OR Technic, Educational OR Technic, Training OR Technics, Educational
OR Technics, Training OR Technique, Educational OR Technique, Training
OR Techniques, Educational OR Techniques, Training OR Training Activities
OR Training Activity OR Training Technic OR Training Technics
OR Training Technique OR Training Techniques OR Training, Academic)

AND

(Medical Student OR Medical Students OR Student, Medical)

## Web of Science

((point of care systems) OR (point of care AND systems) OR (point of care) OR (point AND care))
AND
((diagnostic imaging) OR (diagnostic AND imaging) OR ultrasound OR ultrasonography OR ultrasonics OR ultrasounds)
AND
(Academic Training OR Training Activities OR Training Activity OR Activities, Training
OR Activity, Training OR Educational Technic OR Educational Technics
OR Educational Technique OR Educational Techniques OR Teaching Method
OR Teaching Methods OR Method, Teaching OR Pedagogies
OR Teaching-Learning Process OR Technic, Educational OR Technic, Training
OR Technics, Educational OR Technics, Training OR Technique, Educational
OR Technique, Training OR Techniques, Educational OR Techniques, Training
OR Training, Academic)
AND
(Medical Student OR Medical Students OR Student, Medical)

## Scopus

(point of care systems OR (point of care AND systems) OR (point AND care))
AND
(diagnostic imaging OR (diagnostic AND imaging) OR ultrasound OR ultrasonography OR ultrasonics)
AND
(academic training OR training activities OR training activity OR educational technic
OR educational technics OR educational technique OR educational techniques
OR teaching method OR teaching methods OR pedagogy OR pedagogics
OR teaching-learning process OR training technique OR training techniques
OR training, academic)
AND
(medical student OR medical students OR student, medical)

## Embase

('point of care systems':ti,ab,kw OR ('point of care':ti,ab,kw AND systems:ti,ab,kw)
OR ('point':ti,ab,kw AND 'care':ti,ab,kw))
AND
('diagnostic imaging':ti,ab,kw OR ('diagnostic':ti,ab,kw AND 'imaging':ti,ab,kw)
OR ultrasound:ti,ab,kw OR ultrasonography:ti,ab,kw
OR ultrasonics:ti,ab,kw OR ultrasounds:ti,ab,kw)
AND
('academic training':ti,ab,kw OR 'training activities':ti,ab,kw
OR 'training activity':ti,ab,kw OR 'educational technic':ti,ab,kw
OR 'educational technics':ti,ab,kw OR 'educational technique':ti,ab,kw
OR 'educational techniques':ti,ab,kw OR 'teaching method':ti,ab,kw
OR 'teaching methods':ti,ab,kw OR 'method, teaching':ti,ab,kw
OR 'methods, teaching':ti,ab,kw OR pedagogy:ti,ab,kw
OR pedagogies:ti,ab,kw OR 'teaching-learning process':ti,ab,kw
OR 'training, academic':ti,ab,kw)
AND
('medical student':ti,ab,kw OR 'medical students':ti,ab,kw
OR 'student, medical':ti,ab,kw)

## CINAHL

((MH "Point-of-Care Systems") OR point of care systems OR point of care OR point AND care)
AND
((MH "Diagnostic Imaging") OR diagnostic imaging OR diagnostic AND imaging
OR ultrasound OR ultrasonography OR ultrasonics OR ultrasounds)
AND
((MH "Education, Medical") OR (MH "Teaching Methods")
OR (MH "Educational Technology") OR academic training
OR training activity OR educational technique* OR teaching method*
OR teaching-learning process)
AND
((MH "Students, Medical") OR medical student OR medical students
OR student, medical)

## ERIC

(point of care ultrasound OR POCUS)
AND
(medical education OR medical training OR clinical education)
AND
(ultrasound OR diagnostic imaging OR ultrasonography)
AND
(medical student OR medical students)

## Notes

- Searches were adapted to the syntax and indexing terms required by each database.

- Controlled vocabulary (e.g., MeSH, Emtree, and CINAHL Headings) was combined with free-text terms whenever applicable.

- No language or publication date restrictions were applied.
