# Supplementary material for: Teaching Methodologies and Educational Outcomes of Point‐of‐Care Ultrasound in Undergraduate Medical Education: A Scoping Review
Source: Clin Teach. 2026 Jul 29;23(5):e70485. doi: 10.1111/tct.70485 (PMC13420744; doi:10.1111/tct.70485)
Supplement: Supplementary file 2 — Table S1: Characteristics of included studies on point‐of‐care ultrasound (POCUS) education in undergraduate medical education (n = 59). [file TCT-23-e70485-s002.docx]

Supplementary Table. Characteristics of Included Studies on Point-of-Care Ultrasound (POCUS) Education in Undergraduate Medical Education (n = 59).

| **Study ID** | **Author (Year)** | **Country** | **Participants** | **Sample Size** | **Study Design** | **Educational Intervention / Focus** | **Main Outcomes Evaluated** |
| --- | --- | --- | --- | --- | --- | --- | --- |
| S1 | Abu-Zidan & Cevik (2019) | United Arab Emirates | 5th-year medical students | 88 | Descriptive study | Ultrasound educational experience | Perceptions, educational value |
| S2 | Goodcoff et al. (2019) | United States | 1st–2nd year medical students | 58 | Descriptive survey | Student-led ultrasound curriculum | Satisfaction, engagement |
| S3 | Nagi et al. (2024) | United States | Medical students | 14 studies | Narrative review | Undergraduate ultrasound education | Educational approaches |
| S4 | Blickendorf et al. (2014) | United States | 1st–2nd year medical students | 54 | Program implementation study | Longitudinal curriculum | Feasibility, learner outcomes |
| S5 | Cawthorn et al. (2014) | Canada | 1st and 3rd year medical students | 57 | Comparative cohort study | Peer-assisted ultrasound learning | Knowledge acquisition |
| S6 | Coiffier et al. (2019) | China | Medical students | 221 | Curriculum implementation study | Integrated ultrasound curriculum | Learning experience |
| S7 | DeCara et al. (2005) | United States | 4th-year medical students | 10 | Quasi-experimental study | Echocardiography training | Knowledge and skills |
| S8 | Johnson et al. (2024) | United States | 1st-year medical students | 24 | Prospective observational study | POCUS educational intervention | Knowledge, perceptions |
| S9 | Smith et al. (2018) | United States | Residents and senior students | 30 | Mixed-methods quasi-experimental study | Ultrasound training | Competence and perceptions |
| S10 | Ang et al. (2018) | Australia | 4th–5th year medical students | 27 | Quasi-experimental study | Clinical ultrasound training | Knowledge and confidence |
| S11 | Yamada et al. (2018) | Japan | Medical students | 60 | Quasi-experimental study | Ultrasound education program | Knowledge, image interpretation |
| S12 | Feilchenfeld et al. (2018) | Canada | Published literature | 473 documents | Qualitative discourse analysis | POCUS discourse in medical education | Curricular integration |
| S13 | Zawadka et al. (2019) | Poland | Final-year medical students | 57 | Prospective quasi-experimental study | Ultrasound course | Performance and confidence |
| S14 | Feilchenfeld et al. (2017) | Canada | Published literature | 403 articles | Systematic review and discourse analysis | POCUS curriculum integration | Educational discourse |
| S15 | Ferderber et al. (2022) | United States | 3rd-year medical students | 75 | Quasi-experimental study | Musculoskeletal ultrasound training | Confidence and procedural skills |
| S16 | Amini et al. (2015) | United States | 3rd-year medical students | 95 | Cross-sectional study | Ultrasound curriculum | Confidence and performance |
| S17 | Cook et al. (2020) | United States | 3rd-year medical students | 68 | Quasi-experimental study | Obstetric and gynecologic ultrasound | Knowledge and confidence |
| S18 | Andersen et al. (2014) | Norway | 5th-year medical students | 30 | Prospective observational study | Bedside ultrasound practice | Diagnostic accuracy |
| S19 | Florescu et al. (2015) | Romania | 2nd–6th year medical students | 76 | Randomized controlled trial | Ultrasound teaching methods | Knowledge and practical skills |
| S20 | Hempel et al. (2015) | Germany | 3rd-year medical students | 60 | Randomized controlled trial | Ultrasound education | Knowledge and skills |
| S21 | Meyer et al. (2025) | Germany | Preclinical and clinical students | 318 | Cross-sectional study | Acceptance of POCUS | TAM-based perceptions |
| S22 | Lum et al. (2021) | United States | 3rd–4th year medical students | 62 | Prospective observational study | Simulation-based ultrasound training | Knowledge retention |
| S23 | Kumar et al. (2018) | United States | 1st-year medical students | 40 | Quasi-experimental study | Introductory ultrasound training | OSCE performance |
| S24 | Linehan et al. (2019) | Canada | 2nd-year medical students | 136 | Cross-sectional mixed-methods study | Student radiology interest group | Retention and satisfaction |
| S25 | Nelson et al. (2016) | United States | 1st-year medical students | 142 | Prospective quasi-experimental study | Ultrasound skills curriculum | OSCE performance |
| S26 | Parks et al. (2015) | Canada | Students and residents | 12 | Prospective observational study | Emergency ultrasound simulation | Diagnostic accuracy |
| S27 | Rappaport et al. (2019) | United States | 1st-year medical students | 24 | Longitudinal study | Ultrasound training | Skill retention |
| S28 | Sena et al. (2020) | United States | Not applicable | NA | Curriculum development study | Transition-to-residency curriculum | Curriculum framework |
| S29 | Gun et al. (2022) | Türkiye | Final-year medical students | 60 | Prospective quasi-experimental study | Ultrasound course | Retention after six months |
| S30 | Steller et al. (2014) | United States | Medical students | 9 | Descriptive study | USEFUL protocol training | Feasibility |
| S31 | Webb et al. (2014) | United States | 1st-year medical students | 154 | Cross-sectional study | Introductory ultrasound curriculum | Perceptions and confidence |
| S32 | Talon et al. (2021) | United States | Internal medicine residents | 85 | Quasi-experimental study | Ultrasound curriculum | Knowledge and confidence |
| S33 | Liteplo et al. (2018) | United States | Residents and program directors | 115 | Cross-sectional study | Gamification in ultrasound education | Motivation and perceptions |
| S34 | Olivares-Perez et al. (2022) | United States | 1st-year medical students | 161 | Quasi-experimental study | Ultrasound-assisted anatomy teaching | Knowledge and confidence |
| S35 | Margenfeld et al. (2024) | Switzerland | Published studies | 142 studies | Scoping review | Cadaveric ultrasound education | Educational characteristics |
| S36 | Cheng et al. (2013) | Taiwan | 5th-year medical students | 68 | Prospective observational study | Ultrasound training | Performance and satisfaction |
| S37 | Altersberger et al. (2019) | Austria | 4th-year medical students | 640 | Cross-sectional study | Ultrasound learning methods | Self-efficacy and perceptions |
| S38 | Dickson et al. (2015) | United Kingdom | Postgraduate ultrasound students | 14 | Action research study | Ultrasound learning activities | Educational experience |
| S39 | Nichols et al. (2023) | United States | 1st-year medical students | 32 | Prospective quasi-experimental study | Ultrasound-assisted palpation | Accuracy |
| S40 | de Vries et al. (2018) | United States | 1st-year medical students | 64 | Randomized controlled trial | Ultrasound-assisted palpation | Confidence and accuracy |
| S41 | Hempel et al. (2014) | Germany | Students and physicians | 91 | Prospective observational study | Emergency ultrasound course | Knowledge retention |
| S42 | Dieden et al. (2019) | Sweden | Biomedical science students | 8 | Qualitative study | Ultrasound learning experiences | Student perceptions |
| S43 | Hoppmann et al. (2022) | International | Experts, students and residents | 156 | Delphi consensus study | Undergraduate POCUS recommendations | Consensus competencies |
| S44 | Correia et al. (2023) | South Africa | Human Life Sciences students | 11 | Qualitative study | Ultrasound education experience | Student perceptions |
| S45 | Barth et al. (2024) | Germany | Published studies | 26 studies | Narrative review | Ultrasound educators and teaching roles | Educational roles |
| S46 | Zavitz et al. (2021) | United States | 4th-year medical students | 141 | Quasi-experimental study | Ultrasound intervention | Satisfaction and perceptions |
| S47 | Allen et al. (2023) | United States | Medical students | NA | Commentary | Flipped classroom and self-study | Educational reflections |
| S48 | Ienghong et al. (2023) | Thailand | Emergency medicine residents | 24 | Retrospective observational study | Ultrasound curriculum | Knowledge and satisfaction |
| S49 | Vandenbossche et al. (2023) | Belgium | 2nd-year medical students | 181 | Randomized crossover trial | Ultrasound-enhanced anatomy teaching | Knowledge acquisition |
| S50 | Fuchs et al. (2018) | Israel | 3rd–4th year medical students | 29 | Prospective cohort study | Cardiac ultrasound training | Diagnostic performance |
| S51 | Recker et al. (2023) | Germany | Final-year medical students | NA | Curriculum development study | BI-POCUS curriculum | Curriculum design |
| S52 | Galvez et al. (2020) | Chile | Medical residents | 10 | Mixed-methods study | Educational software/process mining | Usability and learning |
| S53 | Olszynski et al. (2018) | Canada | 2nd-year medical students | 97 | Controlled intervention study | Online ultrasound module | Knowledge acquisition |
| S54 | Nourkami-Tutdibi et al. (2021) | Germany | Medical students | 75 | Experience report | Institutional ultrasound curriculum | Student evaluation |
| S55 | Fernández-Lao et al. (2016) | Spain | Health sciences students | 49 | Randomized controlled trial | Ultrasound educational intervention | Skills acquisition |
| S56 | Rempell et al. (2016) | United States | 1st–2nd year medical students | 214 | Pilot study | Ultrasound educational program | Perceptions |
| S57 | Matschl et al. (2024) | Germany | Published studies | 126 studies | Scoping review | Obstetrics and gynecologic ultrasound education | Educational strategies |
| S58 | Alrahmani et al. (2024) | Saudi Arabia | 5th-year medical students | 54 | Retrospective quasi-experimental study | Ultrasound course | Long-term application of skills |
| S59 | Harthoorn et al. (2024) | Netherlands | Clinical educators | 44 interviews | Qualitative study | Imaging education in medical curricula | Educational priorities |

**Abbreviations:** POCUS = Point-of-Care Ultrasound; OSCE = Objective Structured Clinical Examination; TAM = Technology Acceptance Model; UAE = United Arab Emirates.
